# *EDN1* Gene Variant is Associated with Neonatal Persistent Pulmonary Hypertension

**DOI:** 10.1038/srep29877

**Published:** 2016-07-18

**Authors:** Mei Mei, Guoqiang Cheng, Bijun Sun, Lin Yang, Huijun Wang, Jinqiao Sun, Wenhao Zhou

**Affiliations:** 1 Department of Neonatology, Children’s Hospital of Fudan University, Shanghai, China; 2 Institute of Pediatrics, Children’s Hospital of Fudan University, Shanghai, China; 3 Department of Clinical Immunology, Children’s Hospital of Fudan University, Shanghai, China; 4 Key Laboratory of Neonatal Diseases, Ministry of Health, China

## Abstract

Recent studies have suggested associations between certain genetic variants and susceptibility to persistent pulmonary hypertension of the newborn (PPHN). The aim of the study was to evaluate the association of *EDN1, NOS3, ACE* and *VEGFA* genes with PPHN. Neonates with respiratory distress were enrolled in the study, whose gestational age ≥34 weeks, age ≤3 days. They were divided into PPHN and non-PPHN group. The *EDN1, NOS3, ACE* and *VEGFA* genes were detected by next-generation sequencing, and the results were validated by Sanger sequencing. Serum endothelin-1 (ET-1) levels were quantified by ELISA. A total of 112 neonates were enrolled (n = 55 in PPHN group; n = 57 in non-PPHN group). There is a significantly difference in the genotype distribution of *EDN1* rs2070699 between the PPHN and non-PPHN group (*P* = 0). A higher frequency of the rs2070699 T allele was observed in the PPHN group (54.5% vs 27.2%; OR = 3.89; 95%CI 1.96–7.72). The rs2070699 T allele was associated with higher ET-1 levels (3.333 ± 2.517 pg/mL vs 1.223 ± 0.856 pg/mL; *P* = 0.002) and a longer ventilation period (5.8 ± 2.6 days vs 3.6 ± 3.3 days; *P* = 0). The results suggest there is an association between *EDN1* and PPHN. The presence of the rs2070699 T allele increased the risk of PPHN in neonates with respiratory distress.

Persistent pulmonary hypertension of the newborn (PPHN) occurs when the pulmonary vascular resistance remains elevated after birth, resulting in right to left shunting of blood through fetal circulatory pathways. This, in turn, leads to severe hypoxemia that may not respond to conventional respiratory support. The prevalence of PPHN has been estimated at 1.9 per 1000 live births^1^. PPHN can be easily triggered in newborns by hypoxic lung diseases such as meconium aspiration syndrome, respiratory distress syndrome, and pneumonia.

Recent studies of pulmonary hypertension in adults and neonates have increased our understanding of the genetic basis of the disease. On one hand, genes involved in the transforming growth factor-β superfamily, nitric oxide pathway, potassium channel family and endothelin system have been implicated in pulmonary arterial hypertension and/or PPHN^2^,^3^,^4^. On the other hand, vasoactive substances, including nitric oxide (NO), endothelin-1 (ET-1), angiotensin-II (ANG-II) and vascular endothelial growth factor (VEGF), contribute to progressive changes to pulmonary vasoreactivity via the related genes *NOS3, EDN1, ACE* and *VEGFA*^5^,^6^,^7^,^8^. Endothelial nitric oxide synthase is encoded by *NOS3*^9^. Variants of this gene have been reported to be associated with coronary spasm and hypertension. ET-1 is one of the most potent and long-lasting vasoconstrictors encoded by *EDN1*. It plays an important role in pulmonary arterial hypertension^10^: increased levels of ET-1 were detected in patients with some forms of pulmonary arterial hypertension^11^. The association of *EDN1* gene polymorphisms with hypertension and pulmonary arterial hypertension has been described^12^,^13^. Angiotensin-converting enzyme (ACE) is a key enzyme in the renin-angiotensin system (RAS) and is for the conversion of ANG-I to ANG-II in the circulation, thereby playing a crucial role in blood pressure regulation^14^. It has been suggested that *ACE* gene polymorphisms determine the phenotypic variations of enzyme levels and are associated with essential hypertension^15^,^16^,^17^. *VEGFA* encodes VEGF, a potent endothelial mitogen with angiogenic and vasoactive properties that plays a critical role in lung development^18^. Therefore, the aim of this study was to evaluate the association of *NOS3, EDN1, ACE* and *VEGFA* genes and PPHN.

## Materials and Methods

This study was approved by the ethics committee of the Children’s Hospital of Fudan University and performed from January 2013 to January 2014. The methods were carried out in accordance with the approved guidelines.

### Patients

The population of this study consisted of infants (gestational age ≥34 weeks and birth weight ≥2000 g) who were admitted to the neonatal intensive care unit within 3 days of birth with clinical evidence of respiratory distress.

The inclusion criteria were: Neonates who were born at 34 weeks’ gestation or later, weighed at least 2000g at birth, aged within 3 days, and were admitted to Children’s Hospital of Fudan University with hypoxemic respiratory failure and a requirement for supplemental assisted mechanical ventilation.

The exclusion criteria were: Neonates with structural congenital heart disease excluding patent ductus arteriosus or patent foramen ovale, congenital anomalies such as congenital diaphragmatic hernia.

According to the previous study^3^, PPHN was diagnosed by clinical and echocardiographic data. Clinical criteria consisted of sustained partial pressures of arterial oxygen below 100 mmHg while breathing 100 percent supplemental oxygen, despite mechanical ventilation. Echocardiographic criteria included a structurally normal heart and an elevated pulmonary arterial pressure, that was considered present if there was either right-to-left or bidirectional flow across the patent ductus arteriosus or foramen ovale or a systolic pulmonary arterial pressure greater than or equal to the systemic blood pressure according to Doppler measurement of the tricuspid-regurgitation jet. Infants with respiratory distress who did not have pulmonary hypertension according to these criteria were designated as non-PPHN.

After informed consent was obtained from a parent, infants met above mentioned criteria were divided into two groups: PPHN group and non-PPHN group. Blood sample collection was completed before inhaled nitric oxide administration.

### Candidate gene sequencing by next-generation sequencing (NGS)

Genomic DNA was extracted from peripheral blood leukocytes using a QIAamp DNA Mini Kit (Qiagen, Germany). The DNA concentration was measured using a NanoDrop spectrophotometer (ND-1000, Thermo Fisher Scientific, USA). Genomic DNA samples were tested by NGS sequencing using a custom-designed panel based on an AmpliSeq strategy. The panel, created using the Ion AmpliSeq^TM^ designer software, was designed to identify disease mutations in the following genes: *EDN1, ACE, VEGFA* and *NOS3*. This design allowed for the analysis of 70 exons (*EDN1*: 5, *ACE*: 26, *VEGFA*: 9, *NOS3*: 30) (padding: + /−50 bp). The library was prepared by following the instructions provided by the manufacturers of the kits for fragmentation (Ion Shear, Life Technologies, USA), adaptor and barcode ligation (Ion Xpres Barcode Adapters Kit, Life Technologies, USA) and library quantification (Ion Library Quantification Kit, Life Technologies, USA). We used the Ion OneTouch™ system (Life Technologies, USA) to clonally amplify pooled, barcoded libraries on Ion Sphere™ particles. Torrent Suite™ software was used to compare base calls. NextGENeTM software was used to read alignments and to call variants using the human genomic reference hg19 (NCBI). The variants selected for further analysis met the following criteria: 1) the variant was detected in the sequence reads for both strands, 2) a minimum coverage of 10× was achieved, 3) the variant reads represented >20% of the sequence reads at a particular site, and 4) the targeted region covered all exons and at least 50 bp of all intron/splice sites. The filtered variants were then compared using dbSNP (http://www.ncbi.nlm.nih.gov/projects/SNP/).

### Sanger sequencing

The variants were validated by Sanger sequencing using an automated sequencer (3500XL Genetic Analyzer, Applied Biosystems, USA).

### Serum ET-1 concentration detection

Serum ET-1 levels were measured using an enzyme-linked immunosorbent assay (Endothelin-1 Quantikine ELISA kit, R&D Systems, USA). Each blood sample was collected within 3 days after birth, was placed in a blank tube for 30 minutes, and was then centrifuged at 3000× g for 15 minutes. The serum was stored at −80 °C. Each step was performed according to the manufacturer’s instructions.

### Statistical analysis

#### Sample Size and Power Calculations

We used Quanto 1.4 version (University of Southern California) to calculate the study sample size. Research is an unmatched case-control design study. According to the references, we hypothesized that 40~50% of patients would have a T allele in the baseline, chose inheritance model as Additive (GG, GT, TT) and estimated gene-environment model which would be no interaction between the PPHN and non-PPHN groups. Using a 2-sided α level of 0.05 and preinstalling 0.9976 power for gene to test the futility hypothesis in the analysis, a sample size of 55 patients in the PPHN group and 57 patients in the control group were included finally.

#### Data analysis

Statistical analysis was performed using Stata version 12.0 for Windows (StataCorp). Baseline characteristics were compared between the PPHN and non-PPHN infants using the chi-square test for categorical variables and a Mann-Whitney-Wilcoxon or Kruskal-Wallis rank sumtest for continuous variables. Categorical variables were summarized using frequency and rate, mean and standard deviation for continuous variables. Logistic regression analyses using an α level of 0.05 were performed to determine whether gene expression increased the risk of disease occurrence. The regression imputation was based on a logistic regression model with baseline covariates for gender, gestational age, birth weight, postnatal age, maternal history of disease, and delivery mode.

## Results

### Baseline characteristics of the enrolled infants

In total, 112 infants were enrolled, including 55 neonates in the PPHN group and 57 neonates in the non-PPHN group. There were no significant differences in the baseline characteristics between the PPHN and non-PPHN group (Table 1). Among the infants in the PPHN group, the primary diagnosis were hyaline membrane disease, transient tachypnea of the newborn, perinatal asphyxia, meconium aspiration syndrome, and pneumothorax. Among the infants in the non-PPHN group, the primary diagnose were hyaline membrane disease, transient tachypnea of the newborn, perinatal asphyxia, meconium aspiration syndrome, pneumothorax and pneumonia. There was no significant difference in the primary diagnosis between the PPHN and non-PPHN group (*P* = 0.173) (Table 1). The maximal oxygenation-index values of the PPHN group were higher than non-PPHN group (22.335 ± 23.803 vs 6.961 ± 4.078, *P* = 0). Twenty-five (45.5%) required treatment with inhaled nitric oxide, 28 (50.9%) required vasoactive agents therapy, finally, 5 died of multiorgan failure (9.1%) in PPHN group. While only 1 (1.75%) required vasoactive agents therapy, and none required treatment with inhaled nitric oxide, 1 (1.75%) died finally in non-PPHN group.

**Figure 1:**
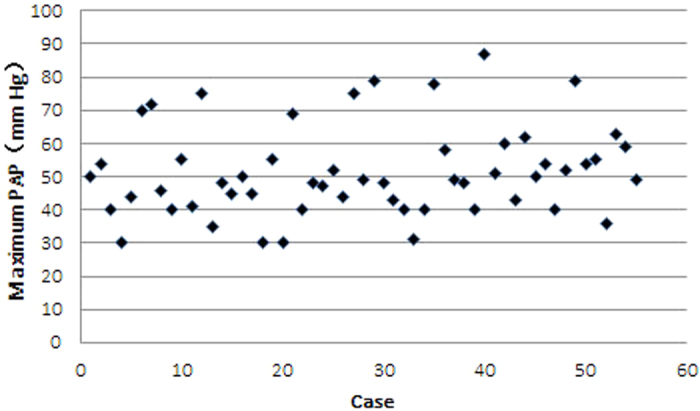
Individual values for maximum pulmonary artery pressure (PAP) in PPHN group.

Although pulmonary artery pressure can be measured directly by Swan Ganz Catheter. There are a number of echocardiographic indicators that help indirectly to measure pulmonary artery pressure (PAP), here we use velocity of the tricuspid regurgitation (TR) jet to assess PAP. The maximum PAP trajectories of each individual patient in PPHN group are visualized in Fig. 1.

### Variants in candidate gene

All the enrolled neonates were evaluated by NGS on the Ion Torrent Personal Genome Machine platform. Although no pathogenic mutations were identified, 48 SNPs were found (Table 2). Thirteen SNPs were found to significantly deviate from Hardy-Weinberg equilibrium in non-PPHN and were therefore excluded for the further analysis. An interesting observation was that differences in the distribution of allele and genotype frequencies of rs2070699 in *EDN1* were observed. A higher frequency of the rs2070699 T allele was observed in the PPHN group (OR = 3.89, 95% confidence interval (CI) 1.96–7.72, *P* = 0) (Table 3). After adjustment for gender, gestational age, birth weight, postnatal age, maternal history of disease, and delivery mode, logistic regression analysis showed that rs2070699 T allele carriers were more likely to be PPHN than G carriers (Table 4). In order to understand the association of this genetic variation and severity of the disease, serum ET-1 levels were measured in 40 patients (n = 15 in PPHN group, n = 25 in non-PPHN group).

### ET-1 concentration

In the cohort of neonates tested for ET-1, there were no significant differences in the baseline characteristics between the sequenced PPHN and non-PPHN infants; ET-1 levels were 2.794 ± 2.634 pg/mL and 2.202 ± 1.731 pg/mL, respectively (*P* = 0.722). The *EDN1* GG genotype was associated with significantly lower ET-1 levels (*P* = 0.002) compared with the GT/TT genotypes. Neonates with the T allele seemed to have higher oxygenation index and longer ventilation time (Table 5).

## Discussion

At birth, the fetal cardiopulmonary system rapidly establishes the lung as the gas exchange organ by decreasing pulmonary vascular resistance and increasing pulmonary blood flow. Pulmonary hypertension can be easily triggered in newborns by hypoxic lung disease, apnea, or other causes. Interestingly, not all neonates requiring intensive care for respiratory failure develop PPHN^1^. The disease is most likely a manifestation of pulmonary vascular maladaptation precipitated by an interaction of environmental and genetic factors. A recent single-center study reported the results of the genotype analysis of 88 neonates with documented PPHN; no differences were noted in most candidate genes, including *BMPR2* and nitric oxide synthase^19^. However, PPHN was significantly associated with genetic variants of corticotropin releasing hormone receptor-1 (*CRHR1*) and CRH-binding protein, as well as with significantly increased 17-hydroxyprogesterone levels.

ET was originally isolated in 1988 from the supernatant of a porcine aortic endothelial cell culture and was demonstrated to be a strong vasoconstrictive peptide^20^. ET has 3 isoforms (ET-1, ET-2, and ET-3) that are translated from 3 independent genes^21^. Although structurally and functionally similar, the expression patterns of the 3 endothelins vary considerably. ET-1 is expressed in several tissues, including endothelial cells and cardiomyocytes, whereas ET-2 and ET-3 are mainly expressed in the gastrointestinal tract and neuronal cells, respectively^22^. ET-1 plays a role in a variety of vascular diseases such as hypertension, arteriosclerosis, and ischemic heart disease. ET-1 levels have been examined as an important risk factor for pulmonary arterial hypertension because plasma ET-1 levels were found to be higher in patients with pulmonary arterial hypertension^23^. DNA sequence variations in the *EDN1* gene locus have been reported to be associated with blood pressure levels and idiopathic pulmonary arterial hypertension^13^,^24^,^25^.

The major objective of the present study was to investigate the association of variations of genes involved in the vasomotor reaction with the development of PPHN in patients with respiratory distress. The major findings of this study were that the rs2070699 SNP in the *EDN1* is associated with a predisposition for PPHN in neonates with respiratory distress. The T allele of rs2070699 SNP may increase the risk of PPHN under respiratory distress.

In a study investigating the susceptibility to high-altitude pulmonary edema, Charu *et al*.^26^ found that the T allele of the rs2070699 SNP was overrepresented in subjects experiencing pulmonary edema. Rankinen *et al*.^12^ showed that the hypertension risk associated with rs5370 was particularly enhanced by an rs2070699 polymorphism. We found that the T allele at rs2070699 was significantly associated with an increased risk of PPHN, higher plasma levels of ET-1 and a longer ventilation time under respiratory distress. We did not find that the genetic variants of *VEGFA, NOS3* and *ACE* significantly contributed to PPHN.

In conclusion, this study provides the first evidence of an association between the *EDN1* rs2070699 SNP and the risk of PPHN in Chinese neonates with respiratory distress. However, the sample size is small; thus, the result requires confirmation in a larger sample. The role of this genetic variant in the pathogenesis of PPHN should be further investigated by functional research to provide a new theoretical basis for disease control and prevention.

## Additional Information

**How to cite this article**: Mei, M. *et al*. *EDN1* Gene Variant is Associated with Neonatal Persistent Pulmonary Hypertension. *Sci. Rep*. **6**, 29877; doi: 10.1038/srep29877 (2016).

## Figures and Tables

**Table 1:** Baseline characteristics of the enrolled infants.

	PPHN (N = 55)	non-PPHN (N = 57)	z/χ^2^	*P*
Male sex (%)	31(56.36)	36(63.16)	0.538	0.463
Gestational age (wk)	37.58 ± 2.06	37.18 ± 2.05	1.197	0.232
Birth weight (g)	2986.1 ± 564.3	2880.6 ± 559.9	1.304	0.192
Age at enrollment (d)	2.4 ± 1.4	2.1 ± 0.9	0.782	0.434
Cesarean section delivery (%)	40(74.07)	42(73.68)	0.002	0.963
Ventilation time (d)*	5.4 ± 3.2	4.3 ± 3.9	3.333	0.001
Maximal oxygenation index*	22.335 ± 23.803	6.961 ± 4.078	6.169	0
Inhaled nitric oxide (%)*^Ψ^	25(45.5)	0	33.354	0
Vasoactive agents therapy (%)*^Ψ^	528(50.91)	1(1.75)	35.246	0
Mortality (%)^Ψ^	5(9.1)	1(1.75))	2.971	0.110
Primary diagnosis*^Ψ^				
Hyaline membrane disease	28	22	7.761	0.173
Transient tachypnea of the newborn	13	21		
Perinatal asphyxia	9	8		
Meconium aspiration syndrome	4	1		
Pneumothorax	1	2		
Pneumonia	0	3		

*P < 0.05 for the comparison between groups. ^Ψ^χ2-Fisher’s Exact test.

**Table 2:** SNPs identified in 112 infants by Ion Torrent sequencing.

Gene	Chr	Nucleotide position	SNP (rs)	Nucleotide change	Minor allele frequency
*ACE*	17	61554632	rs147912715	G/A	0.0028
	17	61556298	rs4295	C/G	0.3666
	17	61556342	rs182340837	A/C	0.0022
	17	61556410	rs13306087	G/A	0.0022
	17	61556429	rs117134739	A/G	0.0034
	17	61557200	rs4298	C/T	0.1444
	17	61557861	rs148882466	C/T	0.0004
	17	61559923	rs4309	C/T	0.4235
	17	61560501	rs28730839	C/G	0.0004
	17	61560763	rs4311	T/C	0.3343
	17	61562309	rs4316	C/T	0.4808
	17	61562553	rs4320	G/A	0.4748
	17	61562774	rs4321	T/C	0.4702
	17	61564052	rs4331	A/G	0.4704
	17	61565990	rs4341	G/C	0.47
	17	61565998	rs4342	A/C	0.47
	17	61566031	rs4343	G/A	0.3568
	17	61573761	rs4362	T/C	0.4125
	17	61574443	rs117135474	C/T	0.0046
	17	61574492	rs4363	G/A	0.4181
*EDN1*	6	12290732	rs10478694	A/-	0.2015
	6	12292599	rs150035515	G/A	0.0026
	6	12292615	rs183694577	G/A	0.003
	6	12292772	rs2070699	G/T	0.3562
	6	12294137	rs1800543	T/C	0.274
	6	12294258	rs5369	A/G	0.0986
	6	12296255	rs5370	G/T	0.2474
	6	12297028	rs9296344	T/C	0.1442
*NOS3*	7	150696008	rs1007311	A/G	0.4591
	7	150692444	rs1800781	G/A	0.1084
	7	150695726	rs1549758	T/C	0.1823
	7	150696111	rs1799983	T/G	0.1763
	7	150698879	rs1800780	A/G	0.4305
	7	150699250	rs1800782	G/T	0.127
	7	150704250	rs2566514	C/G	0.4313
	7	150704400	rs3730305	C/A	0.1396
	7	150706383	rs753482	C/A	0.1857
	7	150706915	rs743506	G/A	0.2831
	7	150708089	rs891512	A/G	0.1116
*VEGFA*	6	43738350	rs2010963	C/G	0.3261
	6	43738977	rs25648	C/T	0.1212
	6	43746169	rs3025000	C/T	0.2354
	6	43748545	rs185218985	G/A	0.0008
	6	43748643	rs3025052	T/C	0.011
	6	43752536	rs3025039	C/T	0.1336
	6	43753051	rs3025040	C/T	0.1512
	6	43753212	rs10434	A/G	0.3476
	6	43753325	rs3025053	G/A	0.0871

**Table 3:** Genotype and allele distribution of rs2070699 in 112 neonates.

	PPHN	non-PPHN	χ^2^	*P*	OR	95% CI
Genotype frequency						
GG	12(21.8)	30(52.6)	11.339	0.001	0.25	0.11, 0.57
GT	26(47.3)	23(40.4)	0.545	0.460	1.33	0.63, 2.80
TTΨ	17(30.9)	4(7.0)	10.488	0.001	5.93	1.85, 19.03
Allele frequency						
G	50(45.5)	83(72.8)				
T	60(54.5)	31(27.2)	31.797	0	3.89	1.96, 7.72

*The Chi-square test was used. Odds ratios and 95% CI were estimated using an additive model. As to *END1* gene, the T allele of rs2070699 was more prevalent in PPHN patients compared to control subjects after Bonferroni-Dunn’s multiple comparisons post-hoc analysis for test groups (0.05/3), there revealed significant differences in frequencies of G/T alleles. CI: confidence interval.

^Ψ^χ2-Fisher’s Exact test.

**Table 4:** Logistic regression analysis of risk factors associated with PPHN Risk factor.

Factor	β-value	Odds Ratio	95% CI		z	*P*
rs2070699 T allele	1.358	3.889	1.96	7.72	3.88	0
Gestational age	0.140	1.150	0.85	1.56	0.91	0.365
Birth weight	−0.000	0.999	0.99	1.00	−0.29	0.769
Gender	0.658	1.931	0.75	5.00	1.35	0.175
Postnatal age	0.272	1.312	0.87	1.98	1.29	0.195
Maternal history	0.072	1.074	0.26	4.36	0.1	0.92
Cesarean section delivery	−0.316	0.728	0.24	2.18	−0.57	0.571
Constant	−7.439	0.000	3.36E-08	10.28	−1.49	0.136

R^2^ = 0.1737, *P* = 0.0013.

**Table 5:** Comparison of the ET-1 levels between the different genotypes.

	GG (N = 15)	GT + TT (N = 25)	z/χ^2^	*P*
ET-1 (pg/ml)	1.223 ± 0.856	3.333 ± 2.517	3.102	0.002
Maximal oxygenation index	6.275 ± 3.587	20.925 ± 23.139	2.900	0.004
Ventilation time (d)	3.6 ± 3.3	5.8 ± 2.6	3.749	0
Mortality (%)	0	3(12)	1.946	0.279

Note: As we believe that T allele carriers were more susceptible to PPHN, infants with the GT and TT genotypes were combined into one group.

## References

[b1] Walsh-Sukys M. C., Tyson J. E., Wright L. L. . Persistent pulmonary hypertension of the newborn in the era before nitric oxide: Practice variation and outcomes. Pediatrics. 105, 14–20 (2000). 10617698 10.1542/peds.105.1.14

[b2] Machado R. D., Aldred M. A., James V. . Mutations of the TGF-beta type II receptor BMPR2 in pulmonary arterial hypertension. Hum Mutat. 27, 121–132 (2006). 16429395 10.1002/humu.20285

[b3] Pearson D. L., Dawling S., Walsh W. F. . Neonatal pulmonary hypertension urea cycle intermediates, nitric oxide production, and carbamoyl-phosphate synthetase function. N Engl J Med. 344, 1832–1838 (2001). 11407344 10.1056/NEJM200106143442404

[b4] Germain M., Eyries M., Montani D. . Genome-wide association analysis identifies a susceptibility locus for pulmonary arterial hypertension. Nat Genet 45, 518–521 (2013). 23502781 10.1038/ng.2581 PMC3983781

[b5] Pepke-Zaba J. & Morrell N. W. The endothelin system and its role in pulmonary arterial hypertension (PAH). Thorax. 60, 443–444 (2005). 15923241 10.1136/thx.2004.031724 PMC1747432

[b6] Pepke-Zaba J., Higenbottam T. W., Dinh-Xuan A. T. . Inhaled nitric oxide as a cause of selective pulmonary vasodilatation in pulmonary hypertension. Lancet. 338, 1173–1174 (1991). 1682593 10.1016/0140-6736(91)92033-x

[b7] Abman S. H. Impaired vascular endothelial growth factor signaling in the pathogenesis of neonatal pulmonary vascular disease. Adv Exp Med Biol. 661, 323–335 (2010). 20204740 10.1007/978-1-60761-500-2_21

[b8] Muller A. M., Gruhn K., Lange S. . Angiotensin converting enzyme (ACE, CD143) in the regular pulmonary vasculature. Pathologe. 25, 141–146 (2004). 15011000 10.1007/s00292-004-0681-x

[b9] Nakayama M., Yasue H., Yoshimura M. . T-786>C mutation in the 5′-flanking region of the endothelial nitric oxide synthase gene is associated with coronary spasm. Circulation. 99, 2864–2870 (1999). 10359729 10.1161/01.cir.99.22.2864

[b10] Price L. C. & Howard L. S. Endothelin receptor antagonists for pulmonary arterial hypertension: Rationale and place in therapy. Am J Cardiovasc Drugs. 8, 171–185 (2008). 18533738 10.2165/00129784-200808030-00004

[b11] Stewart D. J., Levy R. D., Cernacek P. . Increased plasma endothelin-1 in pulmonary hypertension: Marker or mediator of disease? Ann Intern Med. 114, 464–469 (1991). 1994793 10.7326/0003-4819-114-6-464

[b12] Rankinen T., Church T., Rice T. . Effect of endothelin 1 genotype on blood pressure is dependent on physical activity or fitness levels. Hypertension. 50, 1120–1125 (2007). 17938376 10.1161/HYPERTENSIONAHA.107.093609

[b13] Vadapalli S., Rani H. S., Sastry B. . Endothelin-1 and endothelial nitric oxide polymorphisms in idiopathic pulmonary arterial hypertension. Int J Mol Epidemiol Genet. 1, 208–213 (2010). 21537392 PMC3076769

[b14] Crisan D. & Carr J. Angiotensin I-converting enzyme: Genotype and disease associations. J Mol Diagn. 2, 105–115 (2000). 11229513 10.1016/S1525-1578(10)60624-1 PMC1906907

[b15] Rasyid H., Bakri S. & Yusuf I. Angiotensin-converting enzyme gene polymorphisms, blood pressure and pulse pressure in subjects with essential hypertension in a South Sulawesi Indonesian population. Acta Med Indones. 44, 280–283 (2012). 23314967

[b16] Kabadou I. A., Soualmia H., Jemaa R. . G protein beta3 subunit gene C825T and angiotensin converting enzyme gene insertion/deletion polymorphisms in hypertensive Tunisian population. Clin Lab. 59, 85–92 (2013). 23505911 10.7754/clin.lab.2013.111105

[b17] Martinez-Rodriguez N., Posadas-Romero C., Villarreal-Molina T. . Single nucleotide polymorphisms of the angiotensin-converting enzyme (ACE) gene are associated with essential hypertension and increased ACE enzyme levels in Mexican individuals. PLoS One. 8, e65700 (2013). 23741507 10.1371/journal.pone.0065700 PMC3669228

[b18] Lahm T., Crisostomo P. R., Markel T. A. . The critical role of vascular endothelial growth factor in pulmonary vascular remodeling after lung injury. Shock. 28, 4–14 (2007). 17510598 10.1097/shk.0b013e31804d1998

[b19] Byers H. M., Dagle J. M., Klein J. M. . Variations in CRHR1 are associated with persistent pulmonary hypertension of the newborn. Pediatr Res. 71, 162–167 (2012). 22258127 10.1038/pr.2011.24 PMC3718388

[b20] Yanagisawa M., Kurihara H., Kimura S. . A novel potent vasoconstrictor peptide produced by vascular endothelial cells. Nature. 332, 411–415 (1988). 2451132 10.1038/332411a0

[b21] Inoue A., Yanagisawa M., Kimura S. . The human endothelin family: Three structurally and pharmacologically distinct isopeptides predicted by three separate genes. Proc Natl Acad Sci USA 86, 2863–2867 (1989). 2649896 10.1073/pnas.86.8.2863 PMC287019

[b22] Brunner F., Bras-Silva C., Cerdeira A. S. . Cardiovascular endothelins: Essential regulators of cardiovascular homeostasis. Pharmacol Ther. 111, 508–531 (2006). 16457892 10.1016/j.pharmthera.2005.11.001

[b23] Lador F., Soccal P. M. & Sitbon O. Biomarkers for the prognosis of pulmonary arterial hypertension: Holy Grail or flying circus? J Heart Lung Transplant. 33, 341–343 (2014). 24439967 10.1016/j.healun.2013.12.012

[b24] Asai T., Ohkubo T., Katsuya T. . Endothelin-1 gene variant associates with blood pressure in obese Japanese subjects: The Ohasama Study. Hypertension. 38, 1321–1324 (2001). 11751711 10.1161/hy1101.095333

[b25] Jin J. J., Nakura J., Wu Z. . Association of endothelin-1 gene variant with hypertension. Hypertension. 41, 163–167 (2003). 12511547 10.1161/01.hyp.0000043680.75107.cf

[b26] Charu R., Stobdan T., Ram R. B. . Susceptibility to high altitude pulmonary oedema: Role of ACE and ET-1 polymorphisms. Thorax. 61, 1011–1012 (2006). 17071838 10.1136/thx.2006.066019 PMC2121168

